# (Ammine)(carbon­yl)[hydridotris(pyrazol-1-yl-κ*N* ^2^)borato](triphenyl­phosphine-κ*P*)ruthenium(II) chloride dichloro­methane disolvate

**DOI:** 10.1107/S160053681204247X

**Published:** 2012-10-13

**Authors:** Ting-Chuan Hu, Jun-Xlan Ye, Sheng-Ting He, Guan-Ru Chiang, Yih-Hsing Lo

**Affiliations:** a Department of Applied Physics and Chemistry, Taipei Municipal University of Education, Taipei 10048, Taiwan

## Abstract

In the title compound, [Ru(CO)(NH_3_)(C_9_H_10_BN_6_)(C_18_H_15_P)]Cl·2CH_2_Cl_2_, the coordination environment around the Ru^II^ atom is distorted octa­hedral. One of the Ru—N(Tp) [Tp = hydridotris(pyrazol-1-yl)borate] bond lengths is slightly longer than the other two as a result of the influence of the *trans* CO ligand. In the crystal, N—H⋯Cl hydrogen bonds link the complex cations and Cl^−^ anions. π–π inter­actions between the pyrazole rings [centroid–centroid distance = 3.764 (3) Å] are also present.

## Related literature

For general background to complexes with hydrido­tris(pyrazol­yl)borate ligands, see: Alcock *et al.* (1992 ▶); Burrows (2001 ▶); Chen *et al.* (2010 ▶); Lin *et al.* (2008 ▶); Lo *et al.* (2010 ▶); Pavlik *et al.* (2005 ▶); Tong *et al.* (2008 ▶). For related structures, see: Gemel *et al.* (1996 ▶); Slugovc *et al.* (1998 ▶).
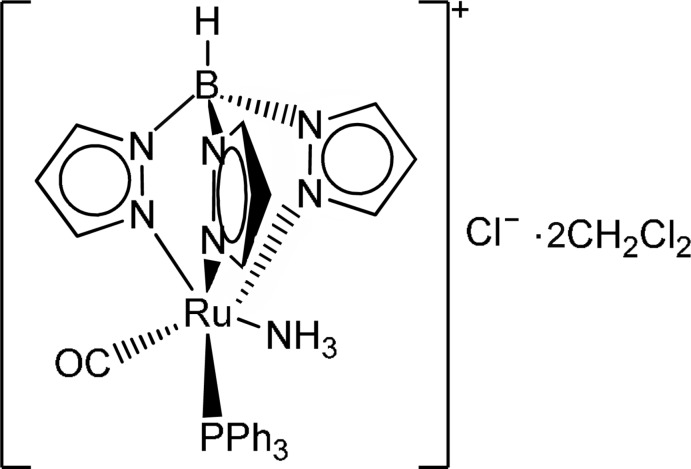


## Experimental

### 

#### Crystal data

[Ru(CO)(NH_3_)(C_9_H_10_BN_6_)(C_18_H_15_P)]Cl·2CH_2_Cl_2_
*M* *_r_* = 826.73
Triclinic, 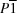

*a* = 12.4813 (4) Å
*b* = 12.5337 (4) Å
*c* = 14.5389 (5) Å
α = 83.520 (1)°
β = 65.602 (1)°
γ = 61.757 (1)°
*V* = 1815.02 (11) Å^3^
*Z* = 2
Mo *K*α radiation
μ = 0.88 mm^−1^
*T* = 200 K
0.19 × 0.18 × 0.06 mm

#### Data collection

Nonuis KappaCCD diffractometer
Absorption correction: multi-scan (*DENZO*/*SCALEPACK*; Otwinowski & Minor, 1997 ▶) *T*_min_ = 0.851, *T*_max_ = 0.949
15492 measured reflections
6218 independent reflections
5363 reflections with *I* > 2σ(*I*)
*R*_int_ = 0.026

#### Refinement

*R*[*F* ^2^ > 2σ(*F* ^2^)] = 0.045
*wR*(*F* ^2^) = 0.119
*S* = 1.06
6218 reflections
414 parameters
H-atom parameters constrained
Δρ_max_ = 1.38 e Å^−3^
Δρ_min_ = −1.29 e Å^−3^

### 

Data collection: *COLLECT* (Nonius, 1998 ▶); cell refinement: *DENZO*/*SCALEPACK* (Otwinowski & Minor, 1997 ▶); data reduction: *DENZO*/*SCALEPACK*; program(s) used to solve structure: *SHELXS97* (Sheldrick, 2008 ▶); program(s) used to refine structure: *SHELXL97* (Sheldrick, 2008 ▶); molecular graphics: *ORTEP-3* (Farrugia, 1997 ▶); software used to prepare material for publication: *WinGX* (Farrugia, 1999 ▶).

## Supplementary Material

Crystal structure: contains datablock(s) I, global. DOI: 10.1107/S160053681204247X/hy2593sup1.cif

Structure factors: contains datablock(s) I. DOI: 10.1107/S160053681204247X/hy2593Isup2.hkl

Additional supplementary materials:  crystallographic information; 3D view; checkCIF report

## Figures and Tables

**Table 1:** Selected bond lengths (Å)

Ru1—N1	2.121 (3)
Ru1—N3	2.136 (3)
Ru1—N5	2.100 (3)
Ru1—N7	2.132 (3)
Ru1—C1	1.851 (5)
Ru1—P1	2.3581 (11)

**Table 2:** Hydrogen-bond geometry (Å, °)

*D*—H⋯*A*	*D*—H	H⋯*A*	*D*⋯*A*	*D*—H⋯*A*
N7—H7*A*⋯Cl1^i^	0.91	2.67	3.454 (4)	145
N7—H7*C*⋯Cl1	0.91	2.46	3.240 (4)	143

Symmetry code: (i) 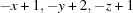.

**Fig. 1.**
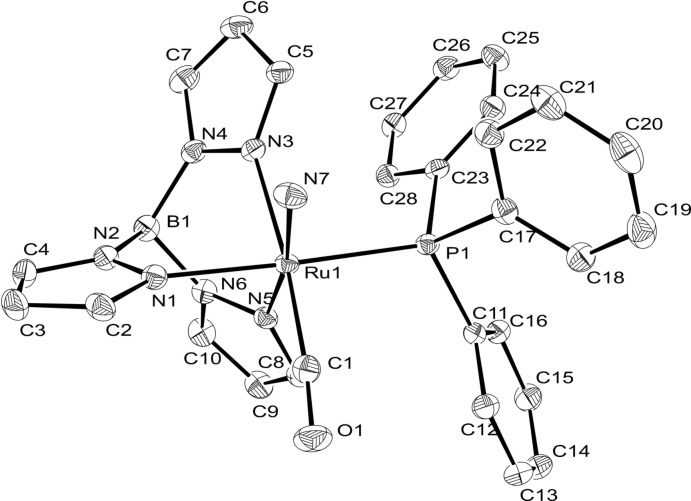
Molecular structure of the title compound. Displacement ellipsoids are drawn at the 30% probability level. H atoms have been omitted for clarity.

## supplementary crystallographic information

### Comment

Ruthenium(II) hydridotris(pyrazolyl)borate complexes, Ru(Tp) [Tp = hydridotris(pyrazol-1-yl)borate], are of interest for stoichiometric and catalytic transformations of organic molecules (Pavlik *et al.*, 2005). The complex RuCl(Tp)(PPh_3_)_2_ (Alcock *et al.*, 1992; Lin *et al.*, 2008) has been used as a starting material for the synthesis of several complexes because of its substitutionally labile chloride and phosphines (Burrows, 2001). The development of Tp chemistry within group VIII has picked up the pace since then (Chen *et al.*, 2010; Lo *et al.*, 2010; Tong *et al.*, 2008).

The title compound was obtained from the reaction of [Ru(Tp)(PPh_3_)(NH_3_)Cl] with CO (Fig. 1). The ν(B—H) vibration of the title complex is found at 2481 cm^-1^, which is characteristic of Tp bound to a metal center in a terdentate (N,N,N) manner. Yellow crystals were obtained by slow diffusion of hexane into a CH_2_Cl_2_ solution at room temperature. The coordination geometry is approximately octahedral. One of the Ru—N(Tp) bond lengths [2.136 (3) Å] is slightly longer than the other two due to the influence of *trans* CO ligand (Table 1) (Gemel *et al.*, 1996; Slugovc *et al.*, 1998). In the crystal, N—H···Cl hydrogen bonds link the complex cations and Cl^-^ anions (Table 2). π–π interactions between the pyrazole rings [centroid–centroid distance = 3.764 (3) Å] are present.

### Experimental

The synthesis of the title compound was carried out as follows. To a solution of [(Tp)(PPh_3_)(NH_3_)RuCl] (0.39 g, 0.45 mmol) in methanol (20 ml), an excess of CO was added. The mixture was heated using a warm water bath for 30 min. A deep yellow color was developed during this time. The reaction mixture was stirred for a further 6 h at room temperature (298 K). Then it was concentrated to approximately half of the volume and cooled to 273 K. The yellow precipitate was filtered off, washed with ethanol and ether and dried under vacuum to give the title compound.

### Refinement

H atoms were placed in idealized positions and constrained to ride on their parent atoms, with C—H = 0.95 (aromatic) and 0.99 (CH_2_), N—H = 0.91 and B—H = 1.00 Å and with *U*_iso_(H) = 1.2(1.5 for ammine)*U*_eq_(C, B, N). The highest residual electron density was found 1.11 Å from Cl4 the deepest hole 0.78 Å from Cl3.

### Crystal data

**Table:**

[Ru(CO)(NH_3_)(C_9_H_10_BN_6_)(C_18_H_15_P)]Cl·2CH_2_Cl_2_	*Z* = 2
*M**_r_* = 826.73	*F*(000) = 836
Triclinic, *P*1	*D*_x_ = 1.513 Mg m^−^^3^
Hall symbol: -P 1	Mo *K*α radiation, λ = 0.71073 Å
*a* = 12.4813 (4) Å	Cell parameters from 6218 reflections
*b* = 12.5337 (4) Å	θ = 1.9–25.0°
*c* = 14.5389 (5) Å	µ = 0.88 mm^−^^1^
α = 83.520 (1)°	*T* = 200 K
β = 65.602 (1)°	Prism, pale brown
γ = 61.757 (1)°	0.19 × 0.18 × 0.06 mm
*V* = 1815.02 (11) Å^3^	

### Data collection

**Table:**

Nonuis KappaCCD diffractometer	6218 independent reflections
Radiation source: fine-focus sealed tube	5363 reflections with *I* > 2σ(*I*)
Graphite monochromator	*R*_int_ = 0.026
Detector resolution: 9 pixels mm^-1^	θ_max_ = 25.0°, θ_min_ = 1.9°
ω and φ scans	*h* = −14→13
Absorption correction: multi-scan (*DENZO*/*SCALEPACK*; Otwinowski & Minor, 1997)	*k* = −14→14
*T*_min_ = 0.851, *T*_max_ = 0.949	*l* = −17→16
15492 measured reflections	

### Refinement

**Table:**

Refinement on *F*^2^	Primary atom site location: structure-invariant direct methods
Least-squares matrix: full	Secondary atom site location: difference Fourier map
*R*[*F*^2^ > 2σ(*F*^2^)] = 0.045	Hydrogen site location: inferred from neighbouring sites
*wR*(*F*^2^) = 0.119	H-atom parameters constrained
*S* = 1.06	*w* = 1/[σ^2^(*F*_o_^2^) + (0.0531*P*)^2^ + 4.644*P*] where *P* = (*F*_o_^2^ + 2*F*_c_^2^)/3
6218 reflections	(Δ/σ)_max_ = 0.002
414 parameters	Δρ_max_ = 1.38 e Å^−^^3^
0 restraints	Δρ_min_ = −1.29 e Å^−^^3^

### Special details

**Table:**

Geometry. All e.s.d.'s (except the e.s.d. in the dihedral angle between two l.s. planes) are estimated using the full covariance matrix. The cell e.s.d.'s are taken into account individually in the estimation of e.s.d.'s in distances, angles and torsion angles; correlations between e.s.d.'s in cell parameters are only used when they are defined by crystal symmetry. An approximate (isotropic) treatment of cell e.s.d.'s is used for estimating e.s.d.'s involving l.s. planes.
Refinement. Refinement of *F*^2^ against ALL reflections. The weighted *R*-factor *wR* and goodness of fit *S* are based on *F*^2^, conventional *R*-factors *R* are based on *F*, with *F* set to zero for negative *F*^2^. The threshold expression of *F*^2^ > σ(*F*^2^) is used only for calculating *R*-factors(gt) *etc*. and is not relevant to the choice of reflections for refinement. *R*-factors based on *F*^2^ are statistically about twice as large as those based on *F*, and *R*- factors based on ALL data will be even larger.

### Fractional atomic coordinates and isotropic or equivalent isotropic displacement parameters (Å^2^)

**Table:**

	*x*	*y*	*z*	*U*_iso_*/*U*_eq_	
C1	0.6168 (4)	0.6084 (4)	0.5701 (3)	0.0318 (10)	
C2	0.8651 (4)	0.6798 (4)	0.4754 (3)	0.0346 (10)	
H2	0.8343	0.6880	0.4238	0.041*	
C3	0.9927 (5)	0.6574 (4)	0.4584 (4)	0.0428 (12)	
H3	1.0637	0.6485	0.3949	0.051*	
C4	0.9938 (4)	0.6510 (4)	0.5529 (4)	0.0386 (11)	
H4	1.0675	0.6358	0.5667	0.046*	
C5	0.5042 (4)	0.9461 (4)	0.8065 (3)	0.0309 (9)	
H5	0.4218	0.9963	0.8010	0.037*	
C6	0.5517 (5)	0.9794 (4)	0.8629 (4)	0.0402 (11)	
H6	0.5096	1.0544	0.9031	0.048*	
C7	0.6727 (5)	0.8808 (4)	0.8484 (4)	0.0406 (11)	
H7	0.7308	0.8755	0.8771	0.049*	
C8	0.6801 (4)	0.4640 (4)	0.7665 (3)	0.0305 (9)	
H8	0.6240	0.4396	0.7566	0.037*	
C9	0.7674 (5)	0.3977 (4)	0.8111 (4)	0.0394 (11)	
H9	0.7830	0.3210	0.8367	0.047*	
C10	0.8270 (5)	0.4663 (4)	0.8105 (3)	0.0375 (11)	
H10	0.8923	0.4450	0.8364	0.045*	
C11	0.3668 (4)	0.6016 (4)	0.7954 (3)	0.0271 (9)	
C12	0.3887 (4)	0.5342 (4)	0.7134 (3)	0.0340 (10)	
H12	0.4084	0.5628	0.6482	0.041*	
C13	0.3819 (5)	0.4265 (4)	0.7261 (4)	0.0406 (11)	
H13	0.3959	0.3819	0.6698	0.049*	
C14	0.3545 (5)	0.3830 (4)	0.8209 (4)	0.0426 (12)	
H14	0.3521	0.3078	0.8293	0.051*	
C15	0.3311 (5)	0.4494 (4)	0.9023 (4)	0.0391 (11)	
H15	0.3115	0.4201	0.9673	0.047*	
C16	0.3356 (4)	0.5595 (4)	0.8905 (3)	0.0322 (10)	
H16	0.3175	0.6056	0.9476	0.039*	
C17	0.2429 (4)	0.8429 (4)	0.7386 (3)	0.0288 (9)	
C18	0.1595 (4)	0.8063 (4)	0.7260 (4)	0.0382 (11)	
H18	0.1697	0.7270	0.7401	0.046*	
C19	0.0618 (5)	0.8856 (5)	0.6930 (4)	0.0453 (12)	
H19	0.0063	0.8596	0.6839	0.054*	
C20	0.0447 (5)	1.0004 (5)	0.6735 (4)	0.0463 (13)	
H20	−0.0211	1.0531	0.6495	0.056*	
C21	0.1227 (5)	1.0402 (4)	0.6886 (3)	0.0418 (11)	
H21	0.1089	1.1209	0.6770	0.050*	
C22	0.2209 (4)	0.9622 (4)	0.7207 (3)	0.0336 (10)	
H22	0.2744	0.9900	0.7309	0.040*	
C23	0.3286 (4)	0.8051 (4)	0.9024 (3)	0.0254 (9)	
C24	0.2045 (4)	0.9035 (4)	0.9547 (3)	0.0319 (9)	
H24	0.1429	0.9404	0.9240	0.038*	
C25	0.1699 (5)	0.9481 (4)	1.0515 (3)	0.0381 (11)	
H25	0.0853	1.0162	1.0862	0.046*	
C26	0.2571 (5)	0.8945 (4)	1.0976 (3)	0.0356 (10)	
H26	0.2323	0.9248	1.1642	0.043*	
C27	0.3814 (4)	0.7963 (4)	1.0463 (3)	0.0314 (9)	
H27	0.4421	0.7595	1.0777	0.038*	
C28	0.4173 (4)	0.7517 (4)	0.9494 (3)	0.0295 (9)	
H28	0.5026	0.6844	0.9146	0.035*	
C29	−0.1096 (9)	0.7309 (9)	0.0766 (6)	0.104 (2)	
H29A	−0.1022	0.6649	0.1218	0.125*	
H29B	−0.2005	0.7998	0.1093	0.125*	
C30	0.6854 (7)	0.1297 (6)	0.5851 (7)	0.091 (3)	
H30A	0.6822	0.1400	0.5176	0.109*	
H30B	0.5930	0.1573	0.6368	0.109*	
N1	0.7924 (3)	0.6881 (3)	0.5748 (3)	0.0286 (8)	
N2	0.8728 (3)	0.6699 (3)	0.6227 (3)	0.0312 (8)	
N3	0.5912 (3)	0.8327 (3)	0.7607 (2)	0.0257 (7)	
N4	0.6956 (3)	0.7934 (3)	0.7871 (3)	0.0292 (8)	
N5	0.6857 (3)	0.5683 (3)	0.7391 (2)	0.0273 (7)	
N6	0.7776 (3)	0.5686 (3)	0.7671 (3)	0.0297 (8)	
N7	0.5307 (4)	0.8656 (3)	0.5813 (3)	0.0340 (8)	
H7A	0.5129	0.9336	0.6141	0.051*	
H7B	0.4554	0.8763	0.5773	0.051*	
H7C	0.5956	0.8519	0.5177	0.051*	
O1	0.6390 (4)	0.5409 (3)	0.5087 (3)	0.0504 (9)	
P1	0.38165 (10)	0.74186 (9)	0.77381 (8)	0.0240 (2)	
Ru1	0.59641 (3)	0.71335 (3)	0.66320 (2)	0.02287 (11)	
B1	0.8199 (5)	0.6693 (5)	0.7388 (4)	0.0322 (11)	
H1	0.8899	0.6545	0.7627	0.039*	
Cl1	0.64723 (12)	0.82746 (10)	0.33609 (8)	0.0368 (3)	
Cl2	0.00232 (16)	0.77728 (14)	0.06739 (14)	0.0696 (4)	
Cl3	−0.0912 (3)	0.6798 (3)	−0.0359 (2)	0.1344 (10)	
Cl4	0.7435 (2)	0.22085 (17)	0.6034 (2)	0.1061 (8)	
Cl5	0.78060 (17)	−0.02459 (14)	0.59197 (12)	0.0695 (4)	

### Atomic displacement parameters (Å^2^)

**Table:**

	*U*^11^	*U*^22^	*U*^33^	*U*^12^	*U*^13^	*U*^23^
C1	0.034 (2)	0.033 (2)	0.027 (2)	−0.018 (2)	−0.010 (2)	0.0062 (19)
C2	0.035 (2)	0.031 (2)	0.026 (2)	−0.014 (2)	−0.005 (2)	0.0019 (18)
C3	0.035 (3)	0.041 (3)	0.035 (3)	−0.019 (2)	0.003 (2)	0.000 (2)
C4	0.028 (2)	0.034 (2)	0.050 (3)	−0.017 (2)	−0.009 (2)	0.004 (2)
C5	0.036 (2)	0.027 (2)	0.027 (2)	−0.0175 (19)	−0.007 (2)	−0.0009 (18)
C6	0.054 (3)	0.032 (2)	0.036 (3)	−0.022 (2)	−0.015 (2)	−0.004 (2)
C7	0.054 (3)	0.045 (3)	0.038 (3)	−0.030 (2)	−0.023 (2)	0.005 (2)
C8	0.029 (2)	0.025 (2)	0.027 (2)	−0.0135 (18)	−0.0020 (19)	0.0007 (17)
C9	0.040 (3)	0.029 (2)	0.039 (3)	−0.014 (2)	−0.013 (2)	0.012 (2)
C10	0.038 (3)	0.039 (3)	0.034 (2)	−0.015 (2)	−0.019 (2)	0.010 (2)
C11	0.024 (2)	0.024 (2)	0.030 (2)	−0.0105 (17)	−0.0073 (18)	−0.0008 (17)
C12	0.036 (2)	0.032 (2)	0.034 (2)	−0.018 (2)	−0.011 (2)	−0.0003 (19)
C13	0.042 (3)	0.035 (2)	0.048 (3)	−0.020 (2)	−0.016 (2)	−0.007 (2)
C14	0.045 (3)	0.028 (2)	0.059 (3)	−0.022 (2)	−0.018 (3)	0.003 (2)
C15	0.040 (3)	0.033 (2)	0.043 (3)	−0.021 (2)	−0.014 (2)	0.011 (2)
C16	0.032 (2)	0.030 (2)	0.035 (2)	−0.0169 (19)	−0.011 (2)	0.0012 (19)
C17	0.028 (2)	0.034 (2)	0.021 (2)	−0.0122 (19)	−0.0081 (18)	0.0002 (17)
C18	0.035 (2)	0.040 (3)	0.040 (3)	−0.017 (2)	−0.015 (2)	−0.002 (2)
C19	0.034 (3)	0.059 (3)	0.043 (3)	−0.017 (2)	−0.020 (2)	−0.004 (2)
C20	0.033 (3)	0.057 (3)	0.034 (3)	−0.008 (2)	−0.017 (2)	0.007 (2)
C21	0.037 (3)	0.038 (3)	0.034 (3)	−0.010 (2)	−0.011 (2)	0.007 (2)
C22	0.033 (2)	0.033 (2)	0.031 (2)	−0.014 (2)	−0.011 (2)	0.0020 (19)
C23	0.032 (2)	0.024 (2)	0.022 (2)	−0.0166 (18)	−0.0079 (18)	0.0012 (16)
C24	0.030 (2)	0.032 (2)	0.032 (2)	−0.0121 (19)	−0.013 (2)	0.0002 (18)
C25	0.033 (2)	0.038 (3)	0.030 (2)	−0.010 (2)	−0.008 (2)	−0.005 (2)
C26	0.042 (3)	0.039 (3)	0.027 (2)	−0.023 (2)	−0.009 (2)	−0.0017 (19)
C27	0.036 (2)	0.037 (2)	0.027 (2)	−0.021 (2)	−0.014 (2)	0.0057 (18)
C28	0.029 (2)	0.028 (2)	0.030 (2)	−0.0131 (18)	−0.0108 (19)	0.0035 (18)
C29	0.091 (6)	0.146	0.088 (6)	−0.076 (4)	−0.019 (5)	−0.008 (5)
C30	0.054 (4)	0.051 (4)	0.174 (8)	−0.020 (3)	−0.054 (5)	−0.003 (4)
N1	0.0305 (19)	0.0272 (18)	0.0270 (19)	−0.0141 (16)	−0.0101 (16)	0.0030 (15)
N2	0.0300 (19)	0.0270 (18)	0.034 (2)	−0.0141 (16)	−0.0098 (17)	0.0030 (15)
N3	0.0307 (19)	0.0263 (18)	0.0230 (17)	−0.0164 (15)	−0.0100 (15)	0.0027 (14)
N4	0.035 (2)	0.0319 (19)	0.0258 (18)	−0.0196 (16)	−0.0123 (16)	0.0008 (15)
N5	0.0277 (18)	0.0252 (18)	0.0234 (18)	−0.0118 (15)	−0.0056 (15)	−0.0002 (14)
N6	0.0329 (19)	0.0297 (19)	0.0264 (18)	−0.0146 (16)	−0.0129 (16)	0.0058 (15)
N7	0.041 (2)	0.0275 (19)	0.0254 (19)	−0.0128 (17)	−0.0101 (17)	0.0016 (15)
O1	0.065 (2)	0.051 (2)	0.0351 (19)	−0.0325 (19)	−0.0113 (18)	−0.0118 (17)
P1	0.0266 (5)	0.0229 (5)	0.0218 (5)	−0.0120 (4)	−0.0084 (5)	0.0000 (4)
Ru1	0.02684 (19)	0.02058 (18)	0.01927 (18)	−0.01138 (14)	−0.00723 (14)	0.00071 (12)
B1	0.033 (3)	0.034 (3)	0.032 (3)	−0.016 (2)	−0.016 (2)	0.005 (2)
Cl1	0.0489 (7)	0.0335 (6)	0.0332 (6)	−0.0221 (5)	−0.0186 (5)	0.0047 (5)
Cl2	0.0614 (9)	0.0593 (9)	0.0913 (12)	−0.0243 (8)	−0.0402 (9)	0.0124 (8)
Cl3	0.214 (3)	0.174 (3)	0.1143 (18)	−0.144 (3)	−0.102 (2)	0.0540 (18)
Cl4	0.0808 (13)	0.0562 (10)	0.186 (2)	−0.0259 (9)	−0.0583 (15)	−0.0194 (12)
Cl5	0.0807 (11)	0.0480 (8)	0.0658 (10)	−0.0308 (8)	−0.0186 (9)	0.0117 (7)

### Geometric parameters (Å, º)

**Table:**

C1—O1	1.155 (5)	C20—C21	1.380 (7)
C2—N1	1.336 (5)	C20—H20	0.9500
C2—C3	1.395 (7)	C21—C22	1.384 (6)
C2—H2	0.9500	C21—H21	0.9500
C3—C4	1.373 (7)	C22—H22	0.9500
C3—H3	0.9500	C23—C24	1.389 (6)
C4—N2	1.348 (6)	C23—C28	1.400 (6)
C4—H4	0.9500	C23—P1	1.838 (4)
C5—N3	1.341 (5)	C24—C25	1.388 (6)
C5—C6	1.383 (6)	C24—H24	0.9500
C5—H5	0.9500	C25—C26	1.376 (6)
C6—C7	1.372 (7)	C25—H25	0.9500
C6—H6	0.9500	C26—C27	1.387 (6)
C7—N4	1.339 (6)	C26—H26	0.9500
C7—H7	0.9500	C27—C28	1.385 (6)
C8—N5	1.349 (5)	C27—H27	0.9500
C8—C9	1.379 (6)	C28—H28	0.9500
C8—H8	0.9500	C29—Cl3	1.710 (8)
C9—C10	1.375 (7)	C29—Cl2	1.704 (8)
C9—H9	0.9500	C29—H29A	0.9900
C10—N6	1.346 (5)	C29—H29B	0.9900
C10—H10	0.9500	C30—Cl4	1.707 (6)
C11—C16	1.388 (6)	C30—Cl5	1.744 (7)
C11—C12	1.396 (6)	C30—H30A	0.9900
C11—P1	1.833 (4)	C30—H30B	0.9900
C12—C13	1.379 (6)	N1—N2	1.367 (5)
C12—H12	0.9500	N2—B1	1.538 (6)
C13—C14	1.390 (7)	N3—N4	1.359 (5)
C13—H13	0.9500	N4—B1	1.543 (6)
C14—C15	1.372 (7)	N5—N6	1.368 (5)
C14—H14	0.9500	N6—B1	1.541 (6)
C15—C16	1.397 (6)	N7—H7A	0.9100
C15—H15	0.9500	N7—H7B	0.9100
C16—H16	0.9500	N7—H7C	0.9100
C17—C18	1.398 (6)	B1—H1	1.0000
C17—C22	1.400 (6)	Ru1—N1	2.121 (3)
C17—P1	1.838 (4)	Ru1—N3	2.136 (3)
C18—C19	1.391 (6)	Ru1—N5	2.100 (3)
C18—H18	0.9500	Ru1—N7	2.132 (3)
C19—C20	1.365 (7)	Ru1—C1	1.851 (5)
C19—H19	0.9500	Ru1—P1	2.3581 (11)
			
O1—C1—Ru1	175.1 (4)	C25—C26—C27	119.7 (4)
N1—C2—C3	110.0 (4)	C25—C26—H26	120.1
N1—C2—H2	125.0	C27—C26—H26	120.1
C3—C2—H2	125.0	C28—C27—C26	120.1 (4)
C4—C3—C2	105.3 (4)	C28—C27—H27	119.9
C4—C3—H3	127.4	C26—C27—H27	119.9
C2—C3—H3	127.4	C27—C28—C23	120.4 (4)
N2—C4—C3	108.6 (4)	C27—C28—H28	119.8
N2—C4—H4	125.7	C23—C28—H28	119.8
C3—C4—H4	125.7	Cl3—C29—Cl2	115.3 (5)
N3—C5—C6	110.1 (4)	Cl3—C29—H29A	108.4
N3—C5—H5	125.0	Cl2—C29—H29A	108.4
C6—C5—H5	125.0	Cl3—C29—H29B	108.4
C7—C6—C5	105.1 (4)	Cl2—C29—H29B	108.4
C7—C6—H6	127.5	H29A—C29—H29B	107.5
C5—C6—H6	127.5	Cl4—C30—Cl5	114.3 (4)
N4—C7—C6	108.9 (4)	Cl4—C30—H30A	108.7
N4—C7—H7	125.6	Cl5—C30—H30A	108.7
C6—C7—H7	125.6	Cl4—C30—H30B	108.7
N5—C8—C9	110.5 (4)	Cl5—C30—H30B	108.7
N5—C8—H8	124.7	H30A—C30—H30B	107.6
C9—C8—H8	124.7	C2—N1—N2	106.9 (3)
C10—C9—C8	105.3 (4)	C2—N1—Ru1	134.2 (3)
C10—C9—H9	127.4	N2—N1—Ru1	118.8 (2)
C8—C9—H9	127.4	C4—N2—N1	109.2 (4)
N6—C10—C9	108.8 (4)	C4—N2—B1	130.9 (4)
N6—C10—H10	125.6	N1—N2—B1	119.9 (3)
C9—C10—H10	125.6	C5—N3—N4	106.6 (3)
C16—C11—C12	118.8 (4)	C5—N3—Ru1	134.6 (3)
C16—C11—P1	122.2 (3)	N4—N3—Ru1	118.7 (2)
C12—C11—P1	119.0 (3)	C7—N4—N3	109.4 (4)
C13—C12—C11	120.7 (4)	C7—N4—B1	130.7 (4)
C13—C12—H12	119.6	N3—N4—B1	119.7 (3)
C11—C12—H12	119.6	C8—N5—N6	106.2 (3)
C12—C13—C14	120.1 (4)	C8—N5—Ru1	135.4 (3)
C12—C13—H13	119.9	N6—N5—Ru1	118.3 (2)
C14—C13—H13	119.9	C10—N6—N5	109.3 (3)
C15—C14—C13	119.6 (4)	C10—N6—B1	129.6 (4)
C15—C14—H14	120.2	N5—N6—B1	120.8 (3)
C13—C14—H14	120.2	Ru1—N7—H7A	109.5
C14—C15—C16	120.7 (4)	Ru1—N7—H7B	109.5
C14—C15—H15	119.7	H7A—N7—H7B	109.5
C16—C15—H15	119.7	Ru1—N7—H7C	109.5
C11—C16—C15	120.0 (4)	H7A—N7—H7C	109.5
C11—C16—H16	120.0	H7B—N7—H7C	109.5
C15—C16—H16	120.0	C11—P1—C23	103.28 (18)
C18—C17—C22	117.9 (4)	C11—P1—C17	103.31 (19)
C18—C17—P1	123.5 (3)	C23—P1—C17	104.22 (18)
C22—C17—P1	118.6 (3)	C11—P1—Ru1	114.55 (13)
C19—C18—C17	120.2 (5)	C23—P1—Ru1	112.47 (13)
C19—C18—H18	119.9	C17—P1—Ru1	117.45 (14)
C17—C18—H18	119.9	C1—Ru1—N5	91.82 (16)
C20—C19—C18	120.8 (5)	C1—Ru1—N1	90.69 (16)
C20—C19—H19	119.6	N5—Ru1—N1	85.37 (13)
C18—C19—H19	119.6	C1—Ru1—N7	92.17 (16)
C19—C20—C21	120.1 (4)	N5—Ru1—N7	171.29 (14)
C19—C20—H20	119.9	N1—Ru1—N7	86.84 (14)
C21—C20—H20	119.9	C1—Ru1—N3	174.48 (16)
C20—C21—C22	119.8 (5)	N5—Ru1—N3	88.26 (13)
C20—C21—H21	120.1	N1—Ru1—N3	83.82 (13)
C22—C21—H21	120.1	N7—Ru1—N3	87.02 (13)
C21—C22—C17	121.2 (4)	C1—Ru1—P1	93.18 (14)
C21—C22—H22	119.4	N5—Ru1—P1	91.56 (9)
C17—C22—H22	119.4	N1—Ru1—P1	175.14 (9)
C24—C23—C28	118.8 (4)	N7—Ru1—P1	95.95 (11)
C24—C23—P1	122.7 (3)	N3—Ru1—P1	92.33 (9)
C28—C23—P1	118.5 (3)	N2—B1—N6	107.9 (4)
C25—C24—C23	120.3 (4)	N2—B1—N4	108.1 (3)
C25—C24—H24	119.8	N6—B1—N4	108.7 (4)
C23—C24—H24	119.8	N2—B1—H1	110.7
C26—C25—C24	120.6 (4)	N6—B1—H1	110.7
C26—C25—H25	119.7	N4—B1—H1	110.7
C24—C25—H25	119.7		

### Hydrogen-bond geometry (Å, º)

**Table:**

*D*—H···*A*	*D*—H	H···*A*	*D*···*A*	*D*—H···*A*
N7—H7*A*···Cl1^i^	0.91	2.67	3.454 (4)	145
N7—H7*C*···Cl1	0.91	2.46	3.240 (4)	143

Symmetry code: (i) −*x*+1, −*y*+2, −*z*+1.
